# Medication use and symptomology in North American women with myalgic encephalomyelitis/chronic fatigue syndrome

**DOI:** 10.3389/fmed.2025.1543158

**Published:** 2025-06-06

**Authors:** Angela Pochakom, Gillian MacNevin, Robyn F. Madden, Amy C. Moss, Julia M. Martin, Sophie Lalonde-Bester, Jill A. Parnell, Eleanor Stein, Jane Shearer

**Affiliations:** ^1^Department of Biochemistry and Molecular Biology, Faculty of Kinesiology, Cumming School of Medicine, University of Calgary, Calgary, AB, Canada; ^2^Department of Health and Physical Education, Mount Royal University, Calgary, AB, Canada; ^3^Metabolic and Cardiovascular Disease Laboratory, University of Alberta, Edmonton, AB, Canada; ^4^Department of Psychiatry, Faculty of Medicine, University of Calgary, Calgary, AB, Canada

**Keywords:** ME/CFS, clinical, drugs, functional capacity, female

## Abstract

**Background:**

There are no known curative treatments for myalgic encephalomyelitis/chronic fatigue syndrome (ME/CFS), and current therapeutic regimens often yield inconsistent results. Despite the profound physical and mental burden experienced by those living with ME/CFS, patients often face a trial-and-error process in finding medications that offer some relief.

**Method:**

The current study surveyed 135 North American women diagnosed with ME/CFS to characterize medication use in relation to disease features, symptomology, and function. Medications were classified into 9 categories according to their primary mechanism of action and therapeutic use.

**Results:**

Participants were primarily middle-aged (47.1 ± 15.3 years) and were diagnosed for a mean duration of 8.4 ± 9.5 years (mean ± SD). Responses showed 68.6% of participants reported taking medications specifically for ME/CFS. Of those taking ME/CFS-related symptom medications, the average use was 3.0 medications per patient, with higher use in US compared to Canadian participants. Analgesic medications (31.7%) were the most frequently used, followed by psychotropic (26.4%), and immune-related medications (10.6%). These trends persisted across different symptom profiles, apart from gastrointestinal associated medication use replacing immune-related medications in those with gastrointestinal, neurological, and psychiatric symptoms. There was no significant correlation found between the number of medications used with disease duration, age, or age at diagnosis. However, a U-shaped relationship between ME/CFS-related symptom medication use and functional capacity as assessed by self-reported physical movement (hours/week) was evident.

**Conclusion:**

Our study highlights the diverse and complex patterns in pharmacological treatment regimens for ME/CFS in women, while also underscoring the need for more tailored and evidence-based therapeutic strategies to address the varied symptom profiles.

## 1 Introduction

Myalgic encephalomyelitis/chronic fatigue syndrome (ME/CFS) is a debilitating medical condition characterized by persistent and severe post-exertional malaise (PEM) accompanied with symptoms related to dysautonomia, cognitive, immune, and endocrine dysfunction (1). ME/CFS can lead to a considerable loss of physical and mental function with many patients becoming house- or bed-bound (2). There is also an established sex-bias in ME/CFS, with the estimated prevalence being 1.5 to 2-fold higher in women than men (3). Furthermore, a recent large-scale study found that being female was associated with greater disease severity, as well as higher reports of symptoms and co-morbidities compared to men (4). Despite this, there are limited treatment options for those with ME/CFS at present. Several factors make it challenging to find an effective treatment for ME/CFS, including limited diagnostic tools, disease heterogeneity, and insufficient awareness among healthcare providers.

There are currently no curative therapies specific to ME/CFS. Rather, management of symptoms is the mainstay of treatment for patients affected by this condition. Guidelines regarding pharmacologic treatments for ME/CFS remain variable and indecisive. Many of these recommendations have not been verified through controlled clinical trials but are rather based on the clinical experience of experts in the field (1). Although there is insufficient supporting evidence for such treatments, individuals with ME/CFS still commonly report using a wide array of medications (5). Furthermore, as several patients are also prescribed medications for other co-morbid illnesses, there is a concern for potential medication interactions that could negatively impact functioning and symptom exacerbation in ME/CFS patients (5).

Thus, the primary objective of this study is to investigate prescription medication use in relation to symptomology, functional capacity, and disease duration in North American women diagnosed with ME/CFS. Taken together, this information will help further characterize medication use among ME/CFS patients, providing foundational insight for future pharmacological investigations.

## 2 Materials and methods

### 2.1 Participants

The study was conducted in accordance with the Code of Ethics of the World Medical Association (Declaration of Helsinki) and approved by the University of Calgary Human Research Ethics Board (Ethics ID: REB20-1941) and Mount Royal University Research Ethics Board (#103049). Participants were recruited through clinic posters, a study-specific social media page, clinics, and clinicians specializing in ME/CFS. Those interested in participating were directed to a link/QR Code to the survey. Participant (i.e., patient or proxy) electronic consent was provided prior to completing the questionnaire. Inclusion criteria for this population-based cross-sectional study was a physician-confirmed diagnosis of ME/CFS. While there is substantial controversy surrounding ME/CFS diagnostic criteria (6–9), the overlapping symptoms include fatigue, cognitive impairment, PEM, sleep disorder, and orthostatic intolerance. Given this, there are core clinical symptoms, and we are confident that physicians were adequately able to diagnose ME/CFS regardless of the exact diagnostic definition used (10, 11).

Exclusion criteria included an inability to complete the online questionnaire (i.e., internet access or language barrier), respondents with identical IP addresses, those that failed to complete >75% of questions, assigned male at birth, minors (<18 y of age), residing outside of North America, as well as individuals with suspected vs. physician confirmed diagnosis of ME/CFS. If the individual was unable to complete the survey themselves, guardians/other family members served as a proxy for responding.

### 2.2 ME/CFS and medication questionnaire

This survey was adapted from a previously published and reliably tested supplement questionnaire (12, 13) and was modified to collect information pertaining to medication, activity, and symptoms in ME/CFS. Initial questions collected demographic information pertaining to age, age at diagnosis, sex, ethnicity, place of residence, and education. In addition, participants were asked to select all the suspected cause(s) or trigger(s) for the participant’s ME/CFS diagnosis from the following options: infection (viral or bacterial), immune system problems, hormonal imbalances, physical and/or emotional trauma, genetic, unknown and/or other. Participants could select more than one primary contributor. To investigate medication use, participants were asked the following 2 questions: (1) Is the participant currently on prescribed or over-the-counter medication(s) for their ME/CFS diagnosis? And (2) Is the participant currently on prescribed or over-the-counter medication(s) for other medical conditions? (Questionnaire in Supplementary material). If participants answered “Yes” to any of the previous questions, they were asked to provide the associated medication identification number(s) (DIN) if located in Canada. Those products without a DIN were manually entered by brand or generic medication name. If elsewhere in North America, brand or generic medication name was entered. Those products not found in medication databases (e.g., foods, supplements, herbal products) were excluded from analysis.

Medications were classified into 9 categories according to their primary mechanism of action and therapeutic use. Psychotropic medications were those that primarily acted on the central nervous system to exert effects on behavior, mood, and perception, including antidepressants [e.g., selective serotonin reuptake inhibitors (SSRIs), serotonin-norepinephrine reuptake inhibitors (SNRIs), tricyclic antidepressants (TCAs)], antipsychotics (e.g., atypical), CNS stimulants (e.g., atypical dopamine reuptake inhibitors, amphetamines), and anxiolytics (e.g., benzodiazepines). The analgesic group comprised of medications primarily utilized for pain relief, including non-steroidal anti-inflammatory medications (NSAIDs), opioids, and low-dose naltrexone. Respiratory medications were those used to mainly treat respiratory conditions (e.g., leukotriene receptor antagonists and bronchodilators), gastrointestinal (GI) medications targeted the gastrointestinal system (e.g., proton pump inhibitors, laxatives, histamine H2-receptor antagonists, anti-emetics), and musculoskeletal (MSK) medications worked specifically on this system (e.g., skeletal muscle relaxants). Immune medications were classified as medications that modulate or regulate immunological processes, including corticosteroids, anti-viral medications, antihistamines, disease-modifying antirheumatic medications. Hormonal agents were those involved with hormone regulation (e.g., thyroid, hormone replacements, estrogens, progestins). Cardiovascular (CV) medications had a primary role in managing cardiovascular health, notably the regulation of blood pressure (i.e., beta-blockers, ACE-inhibitors). Metabolic medications comprised of those used to modulate metabolic pathways, including antidiabetic medications (e.g., incretin mimetics). The specific route, dosing, and timing of administration was not considered. Only medications for ME/CFS-related symptoms were considered in this analysis, although the total number of medications is also reported for context.

### 2.3 Symptom questionnaire

To assess symptom-specific characteristics, participants were asked to identify their top 5 most bothersome clinical symptoms from the following 9 categories: (1) No symptoms, (2) Heart Problems (Cardiovascular), (3) General Symptoms, (4) Neurological Problems, (5) Hormonal Problems, (6) Musculoskeletal (MSK), (7) Gut Problems (GI), (8) Psychiatric Problems, and (9) Other. Within these categories, a total of 38 symptom descriptions were provided (Supplementary material).

### 2.4 Functional capacity

To obtain information regarding functional capacity, hours of physical movement were explored (e.g., activities of daily living, gardening, housework, grocery shopping, walking, stretching, yoga, etc.). The relationship between physical movement with symptom management was also queried. Specific questions in this domain are available in the data supplement. For the purposes of this study, the number of steps (when available via wearables, e.g., Apple™ or Garmin™ watch) and hours of physical movement per week was used. However, not all participants had access to a device.

### 2.5 Other

The questionnaire was self-report based and was translated into 8 languages for enhanced inclusion (e.g., English, French, Mandarin, Finnish, Hindi, German, Portuguese, Spanish). The questionnaire was hosted online by the Qualtrics^XM^ Survey Platform (Seattle, WA) and was composed of 54 total questions. Section one collected demographics, diagnostic data, and information pertaining to symptoms and pharmaceutical use. The second section gathered movement engagement information and how movement affects patient symptoms. Section three compiled data on dietary supplement use while section four outlined dietary restrictions and special dietary needs. Finally, section five explored patient-physician communication and alternative therapeutic strategies implemented among patients to manage their ME/CFS symptoms. Data from sections 3, 4 and 5 regarding diet, alternative, and complementary health practices, as well as patient-physician communication has been published elsewhere (in preparation).

### 2.6 Statistical analysis

Questions pertaining to participant characteristics (e.g., age, sex, residency, education, etc.), as well as general and symptom-specific medication use (e.g., frequency, types, etc.) are based on descriptive statistics and frequency analysis. Data are reported as mean ± SD unless otherwise specified. Comparison of total and ME/CFS-related symptom medication use between participants in the United States and Canada was performed using a Mann–Whitney test. To assess the strength of association between ME/CFS-related symptom medication use with disease characteristics (i.e., disease duration, age, age at diagnosis), a Spearman’s rank correlation coefficient was utilized, given that the data did not follow a Gaussian distribution. To explore functional capacity, the Kruskal-Wallis test was used to assess if total and ME/CFS-related symptom medication use differed between groups of various physical movement levels. For all tests, significance was set at *p* < 0.05. All statistical analysis was performed on the GraphPad Prism (v 10.3.1) software (GraphPad Software Inc., San Diego, California, USA).

## 3 Results

### 3.1 Participant characteristics

Of a total of 295 responses, 135 eligible responses met the specified inclusion criteria and were subsequently used for analysis in this study. The primary reason for exclusion was residence outside of North America (146/295). North American exclusivity was chosen due to similar drug regulatory environments, medication availability, disease epidemiology, economic factors, as well as language barriers that existed in language translations.

Most participants identified as Caucasian (90%), middle-aged (mean age 47) women. Canada was indicated as the country of residence for the majority of participants (66%), the remaining in the United States. Other descriptive and demographic characteristics are outlined in Table 1.

**Table 1:** Participant characteristics.

Demographic information		Mean (± SD) or Frequency (%)
Participant characteristics	Present age (years)	47.1 ± 15.3
	Age of diagnosis (years)	38.7 ± 12.3
	Duration of ME/CFS (years)	8.4 ± 9.5
Country of residence (*N*)	Canada	90 (66.7)
	United States	45 (33.3)
Ethnicity (*N*)	Caucasian	121 (89.6)
	Asian/Pacific Islander	6 (4.4)
	Hispanic/Latino	3 (2.2)
	Multiracial	5 (3.7)
Highest education (*N*)	High school	20 (14.8)
	Trade, Technical, Vocational	8 (5.9)
	College Diploma	22 (16.3)
	University Undergraduate Degree	49 (36.3)
	Advanced/Professional Degree	33 (24.4)
	Other	1 (0.7)
	Prefer not to disclose	2 (1.5)
Disease characteristics		
Primary symptoms^1^	Post-exertional malaise (physical/emotional)	114 (84.4)
	Difficulty with Memory/Concentration/Focus	91 (67.4)
	Sleep that is not refreshing	78 (57.8)
	Decline in social/work/educational activities	59 (43.7)
	Unexplained muscle and/or joint pain	49 (36.3)
	Exercise intolerance	40 (29.6)
	Dizziness from sitting/lying to standing/sitting	31 (23.0)
	Temperature Instability (heat/cold intolerance)	28 (20.7)
	Headaches/Migraine	28 (20.7)
	Weakness	23 (17.0)

1 Descriptive and frequency statistics of participants. A total of 38 symptoms were listed, participants could also select ‘other’ for those not listed.

In total participants selected their top 5 symptoms. Only the top 10 symptoms are shown.

### 3.2 ME/CFS diagnosis and symptoms

**Figure 1:**
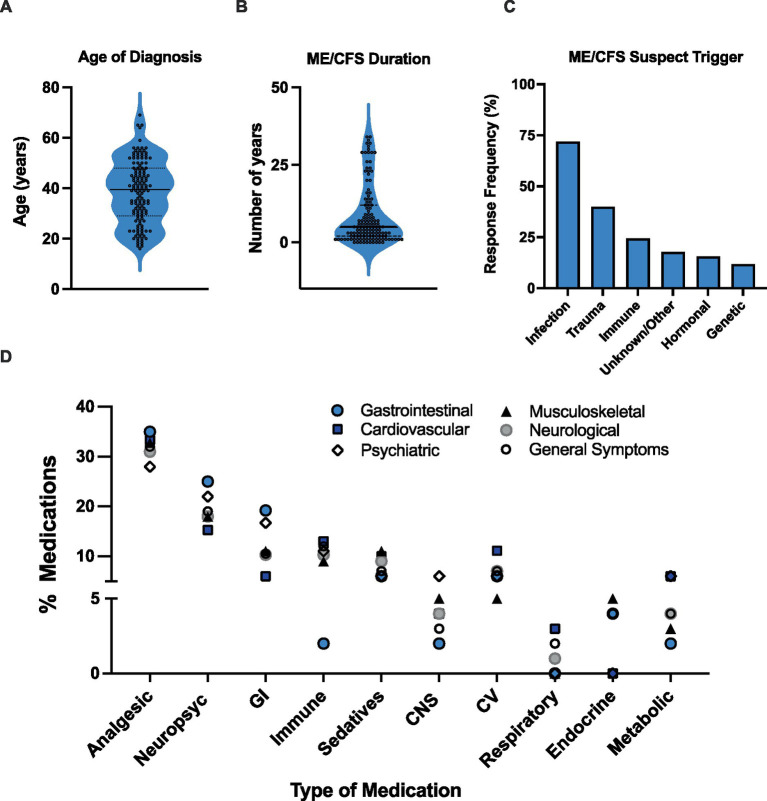
Participant characteristics and medication use. **(A)** Participant age of diagnosis. **(B)** ME/CFS duration. **(C)** Self-reported suspected ME/CFS trigger. Data represents percentage of participants (*N* = 135) choosing a selected trigger. Participants could select more than one primary contributor, with a total of 245 responses recorded. **(D)** Medications specifically prescribed for ME/CFS used among participants with different symptoms. Symptoms were broken down into 6 different categories: Cardiovascular, Neurological, Musculoskeletal, Gastrointestinal, Psychiatric, and General symptoms. Medication category: CV, cardiovascular; GI, gastrointestinal; MSK, musculoskeletal; Psych, psychotropic.

Results showed that participants have been living with a diagnosis of ME/CFS for an average of 8.4 years, with the average age at diagnosis being 39 years old (Figures 1A,B). Participants reported various suspected trigger(s) or cause(s) for their ME/CFS diagnosis including infectious, immunological, hormonal, trauma (physical and/or emotional), genetic, and other/unknown. A total of 245 responses were recorded. Almost three quarters of the sample (71.9%) reported that an infection was the assumed cause for their ME/CFS diagnosis followed by physical and/or emotional trauma (40%) (Figure 1C).

Participants were asked to report on their most bothersome clinical symptoms associated with ME/CFS. Post-exertional malaise (84.4%), difficulty with memory/concentration/focus (67.4%), unrefreshing sleep (57.8%), decline in social/occupational/educational/personal activities (43.7%), and unexplained muscle and/or joint pain (36.3%) were the most frequently indicated (Table 1).

### 3.3 General prescription medication use

Out of the eligible responses, 102 (75.6%) participants indicated that they were taking prescription medication with an average number of 4.2 total medications used per person (Table 2). Notably, medication use for those residing in the United States was statistically greater than those respondents in Canada with 4.9 and 3.8 total medications reported, respectively. Of the total medications, 68.6% reported prescription use for ME/CFS. Like total medication use, ME/CFS-related symptom medications were also greater in US participants with 3.7 and 2.6 medications reported (Table 2). The type of medications prescribed most frequently for ME/CFS, along with prevalent examples in each category, were analgesic (31.7%, NSAIDS, naltrexone), psychotropic (26.4%, SSRIs, SNRIs), and immune-related (10.6%, corticosteroids, anti-virals, antihistamines) medications (Table 2). Although the type and number of medications tended to be slightly different in US participants, this data was underpowered to be statistically evaluated and is therefore not reported.

**Table 2:** Medication use.

Medication use	Mean ± SE
Number of total medications^#^	4.2 ± 0.3
United States (*n* = 35)	4.9 ± 0.5*
Canada (*n* = 67)	3.8 ± 0.3
Number of ME/CFS-related symptom medications^2^	3.0 ± 0.3
United States (*n* = 23)	3.7 ± 0.7
Canada (*n* = 47)	2.6 ± 0.3
Medication class	Frequency (%)
General prescribed medication^1^	102 (75.6)
Medication prescribed specifically for ME/CFS^2^	70 (68.6)
Analgesic	66 (31.7)
Psychotropic	55 (26.4)
Immune-related	22 (10.6)
GI-related	21 (10.1)
Musculoskeletal	17 (8.2)
Cardiovascular	15 (7.2)
Hormonal	8 (3.8)
Respiratory	3 (1.4)
Metabolic	1 (0.5)

1 Descriptive and frequency statistics for medication use and class among participants. This category was evaluated in comparison to all eligible participants (*N* = 135).

2 Only those who reported ME/CFS-specific medication use were included in these categories.

^#^Due to polypharmacy, numbers are not exclusive. Calculation for total medications also include those for ME/CFS. GI, gastrointestinal. *p < 0.05 between US and Canadian participants.

### 3.4 Medications associated with disease characteristics

The relationships between the use of medication with disease duration and age was evaluated. There was no significant correlation found between the length of time with a diagnosis and the number of medications used for ME/CFS-related symptom control (*p* = 0.90; *r* = 0.013). Furthermore, no significant correlation was found between age (*p* = 0.25; *r* = −0.012) or the age at diagnosis (*p* = 0.074; *r* = −0.19) and the number of ME/CFS-related symptom medications used.

Medication use was parsed out among the following clinical symptom categories: (a) Cardiovascular (CV), (b) Neurological, (c) Musculoskeletal (MSK), (d) Gastrointestinal (GI), (e) Psychiatric, and (f) General Symptoms (Figure 1D). Analgesic (27.8–34.6%) and psychotropic (22.2–33.3%) medications consistently ranked as the top two most frequently used across all symptom categories (Figure 1D). Interestingly, the third most frequent medications appear to be either immune-related medications, in individuals experiencing CV (12.5%), neurological (10.3%), MSK (10.9%), and general symptoms (11.6%), or GI-related medications in individuals with neurological (10.3%), GI (19.2%), and psychiatric (16.7%) symptoms (Figure 1D). MSK medication use (12.5%) was also equal to immune-related medications in individuals with CV symptoms (Figure 1D).

**Figure 2:**
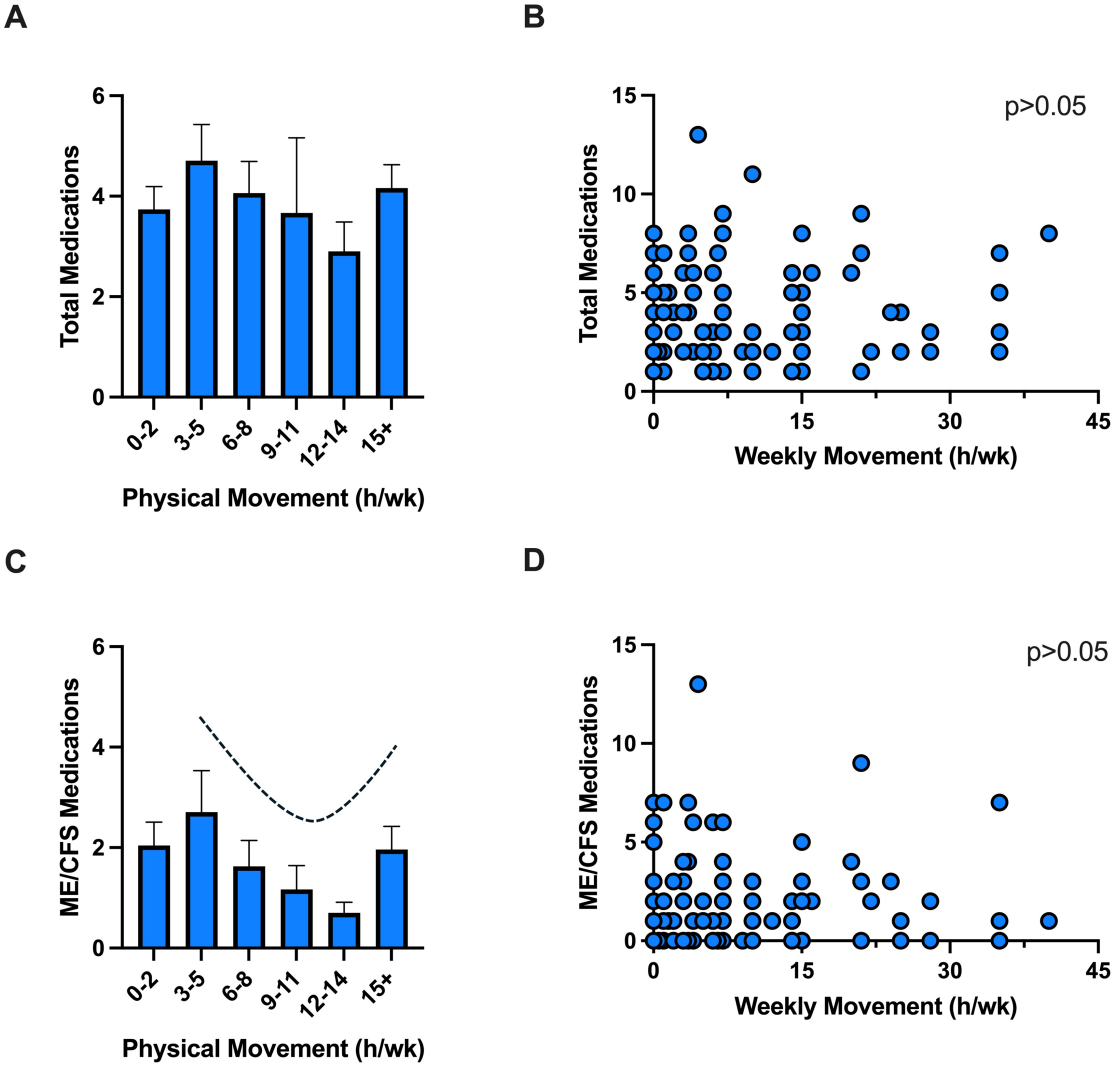
Medication use and physical movement. Total **(A,B)** and ME/CFS **(C,D)** medications use among individuals with various physical movement levels. Individual data showed no correlation between medication use and physical activity (*p* > 0.05). Physical movement was self-reported as hours of physical movement in a typical week (e.g., activities of daily living, gardening, housework, grocery shopping, walking, stretching, yoga, etc.). h, hours; wk., week.

A considerable reduction in activity relative to pre-illness levels and increase in PEM are core characteristics of ME/CFS (14). Thus, medication use in relation to physical movement was assessed as a proxy for functional capacity. There were no significant differences (*p* > 0.05) in total or ME/CFS-related symptom medication use when data was stratified by number of steps (<1,000, 1,000–4,999, >5,000 per day, data not shown) or total hours of movement (Figures 2A,B). However, a U-shaped relation was seen in ME/CFS-related symptom medication use and physical movement (hours/week) (Figures 2C,D). Specifically, ME/CFS-related symptom medication use declined with increasing physical movement with the lowest levels reported with 12–14 h of movement per week (2.9 ± 1.8 medications). Conversely, exceeding this level of movement was associated with increased medication use (>15 h/week = 4.2 ± 2.3 medications).

## 4 Discussion

ME/CFS is a severe, debilitating illness affecting millions of people worldwide, with women being 1.5 to 2 times more likely to be diagnosed than men (3). It is marked by persistent, unexplained fatigue lasting over 6 months and is often accompanied by a complex array of symptoms linked to neurological, autonomic, immune, and endocrine dysfunction (1). Alarmingly, the level of disability caused by ME/CFS frequently exceeds that of other chronic conditions, including multiple sclerosis and cancer (15, 16).

Despite the profound negative impact of this condition on patient quality of life, progress in developing effective therapeutic interventions for ME/CFS has been severely hindered by the lack of reliable diagnostic tools and the wide heterogeneity of symptoms. In fact, there are no known medications that effectively cure ME/CFS, rather the hallmark of treatment is symptom relief (17). As it is common for ME/CFS patients to experience a multitude of symptoms, it often leads to polypharmacy, the potential overuse of medications, and/or negative interactions between medications. Moreover, the efficacy of pharmacological treatments for ME/CFS remain variable in the literature, with insufficient evidence supporting commonly prescribed interventions (18). Such variability makes it challenging for physicians to determine which medications would offer the most benefit. Thus, the current study aimed to further characterize prescription medication use in women diagnosed with ME/CFS in North America, focusing on disease symptomology, functional capacity, and disease duration.

The majority of patients in our study reported taking medication in general, with 68.6% taking medications specifically for ME/CFS symptom management. On average, patients report regularly using 4.2 medications of which 3.0 were for ME/CFS related symptoms, which is below the cut-off of 5 often used to define polypharmacy (19). There was also no association found between ME/CFS-related symptom medication use with disease duration or age, which differs from findings in other chronic conditions, like rheumatoid arthritis, where older age and longer disease duration are found as significant predictors for the increased use of medications (20). Worth mentioning, total and ME/CFS-related symptom medication use among US participants was greater than those respondents from Canada. This fits with existing data and is likely due to differences between the countries in prescription drug regulations, costs, accessibility, and prescribing patterns (21).

The most prescribed medications among our participants were analgesic medications, followed by psychotropic, and immune-related medications. Analgesic medications were also reported as the most used treatments across the majority of ME/CFS symptom categories. These findings are similar to population studies in the US that found that pain relievers were the most frequently used medications in a ME/CFS compared to non-fatigued comparators (22, 23). There may be multiple reasons as to why the use of analgesics is common among ME/CFS patients. Firstly, muscle aches and joint pain are highly prevalent in ME/CFS, in addition being the fifth most bothersome clinical symptom in our study cohort. Analgesics are often the first-line medications used to target such symptoms (24). Furthermore, a recent study reported that pain severity increases following exercise in people with ME/CFS, validating pain as an important component of PEM, the highest reported primary symptom among our patients (25). Thus, we can extrapolate that most patients who experience PEM would also require pharmacological pain management to help maintain daily function. In fact, around 70% of people with ME/CFS meet the diagnostic criteria for fibromyalgia, which is a primary pain condition. Overall, it is evident that analgesics play a central role in ME/CFS symptom management, likely due to the negative impact of pain on functional status and quality of life in this population (26).

The use of psychotropic medications was also widespread in our study, potentially highlighting the considerable impact of depression and psychological stressors on ME/CFS patients. Another aspect may be the historical tendency to characterize ME/CFS as a psychiatric disorder rather than a physical disease, which has frequently led to the prescription of antidepressants as a primary treatment (17). Some patients in our study have lived with an ME/CFS diagnosis for up to 34 years, and since awareness of the condition’s non-psychiatric origins are relatively recent, the numerous antidepressant prescriptions may be a result of former views held by physicians. This is potentially concerning, given the inconclusive evidence regarding the effectiveness of antidepressants for treating ME/CFS specifically. For instance, some clinical trials have reported that SSRIs are ineffective and may even worsen fatigue in ME/CFS patients (27, 28). Additionally, emerging evidence suggests that central serotonin hyperactivity may play a role in the pathogenesis of ME/CFS, which contrasts the low levels of serotonin activity typically seen in depressive disorders (29). As such, it is reasonable to question the use of antidepressants to treat the core symptoms of ME/CFS, and current recommendations do not support their use in patients without depressive symptoms (30).

Immune- or GI-related medications were the third-most utilized medications among the ME/CFS symptom groups. Dysregulation or an abnormal response of the immune system, specifically leading to inflammatory sequelae, is a predominant hypothesis in ME/CFS pathophysiology (31) although no specific pathogen has been identified as a cause. Viral pathogens are often discussed as the origin of this process through the activation of antiviral immune responses, thus triggering systemic inflammation (31). In fact, almost three quarters of our patients self-reported that they believed their condition to be triggered by infection. Given this, it is fitting that immune-related agents, often consisting of corticosteroids (i.e., to target inflammation) and anti-viral medications, were frequently used in our patient cohort.

Our findings regarding GI medications are also noteworthy, as these medications ranked third not only among patients with GI symptoms, but also in those with neurological and psychiatric symptoms. As GI disorders and psychological comorbidities often go hand-in-hand (32), this may have resulted in an increased use of GI-related medications to aid with targeted symptom control. These findings touch upon the concept of the gut microbiome-brain axis, a communication network between the gut microbiome and the enteric and central nervous systems, leading to bidirectional modulation of homeostasis (32). In fact, gut dysbiosis and alteration of gut-brain axis communication is a budding hypothesis for the pathogenesis of ME/CFS (33).

While there was no statistically significant association between physical movement (i.e., a proxy for functional capacity) and ME/CFS-related symptom medication use, a U-shaped relationship was observed. Of note, this included activities of daily living (gardening, housework, grocery shopping, walking) and not necessarily overt exercise (although this was also counted in this metric). Reductions in ME/CFS-related symptom medications were found up to 14 h of physical movement per week. This trend was reversed in those exceeding 15 h of physical movement per week. Since physical activity is well-documented to improve depressive symptoms and mental health in a dose-dependent manner (34), future studies should investigate whether this relationship applies to individuals with ME/CFS, who experience PEM following activity. Current evidence underscores the need for an individualized and cautious approach to physical movement and activity in ME/CFS, emphasizing energy conservation techniques and pacing rather than standardized exercise regimens (35). As such, our findings point toward an optimal physical activity threshold, wherein surpassing this level may exacerbate symptoms for ME/CFS patients. Further research is needed to establish safe and effective strategies tailored to the unique needs of this population, ensuring that therapeutic interventions do not inadvertently harm patients by exacerbating their symptoms.

We would like to acknowledge certain limitations of our study. First, medication use was self-reported, which may have been challenging for participants to identify medications that were for ME/CFS-related symptoms versus co-morbid conditions. Utilizing an objective third-party, such as charts, research nurse, or clinician, to characterize medications and doses could improve the accuracy of our medication data. Furthermore, the study followed a cross-sectional design, in which data was taken at one point in time. This is less representative of the chronic nature of ME/CFS and limited our ability to capture trends in medication use, symptom severity, and functional capacity over time. Lastly, due to limitations in sample size (*N* = 135), we were inclined to categorize medications more broadly, and thus are unable to make any inferences on specific medication classes (i.e., NSAIDs vs. opioids).

## 5 Conclusion

This study provides insight into prescription medication use among women diagnosed with ME/CFS in North America, a complex and debilitating illness that currently has no universally accepted pharmacological treatment. Novel findings show that North American women with the condition take on average 3.0 ME/CFS-related symptom medications per patient. This is in addition to other prescribed medications. This is a valuable piece of information for primary care physicians working to manage the condition in their patients. Further, we show that age of diagnosis and disease duration do not impact medication use in this population. This study also confirms the prevalent use of analgesic medication among patients that suggests pain management is a major factor in addressing ME/CFS symptoms. This is especially important given the relationship between pain and PEM, a defining feature of ME/CFS. Moreover, the use of antidepressants for ME/CFS should be questioned due to limited evidence of benefit and/or potential harm. Additionally, the frequency of immune- and GI-related medications may reflect emerging evidence regarding the role of immunological dysregulation and the gut microbiota-brain-axis in the pathophysiology of ME/CFS. Lastly, approaches to physical movement as a therapeutic strategy should be individualized to prevent over-exertion and symptom exacerbation in ME/CFS patients. While our findings emphasize a strong reliance on pharmacological treatment, they also highlight the need for more targeted and evidence-based therapeutic regimens. Future research should explore the efficacy of specific medications, the underlying mechanisms of ME/CFS, and aim to develop more tailored approaches to patient care.

## Data Availability

The datasets presented in this article are not readily available because of patient confidentiality. The original contributions presented in the study are included in the article, further inquiries can be directed to the corresponding authors. Requests to access the datasets should be directed to jshearer@ucalgary.ca.
