# Supplementary material for: Medication use and symptomology in North American women with myalgic encephalomyelitis/chronic fatigue syndrome
Source: Front Med (Lausanne). 2025 Jun 6;12:1543158. doi: 10.3389/fmed.2025.1543158 (PMC12179203; doi:10.3389/fmed.2025.1543158)
Supplement: Supplementary file 1 [file Data_Sheet_1.pdf]

Supplementary Material

Myalgia Encephalomyelitis/Chronic Fatigue Syndrome Nutrition/Medication Study Questionnaire

Preamble

This survey was adapted from a previously published and reliably tested supplement questionnaire (1,2) and was modified to collect information pertaining to medication, activity, dietary supplement use, special dietary needs and physician communication in Myalgia Encephalomyelitis/Chronic Fatigue Syndrome (ME/CFS).

Introduction

The purpose of the study is to examine the various types of supplements used, dietary patterns followed, and other therapeutic treatment strategies used among patients diagnosed with ME/CFS. This study will assess the information available to participants regarding the health risks of supplements, and to gain an appreciation for factors influencing use, knowledge and adherence to supplements.

If you volunteer to participate in this study, the researcher will ask you to do the following: Step 1 – Inclusion Criteria: In order to participate in the study, the participant must have been formally diagnosed by a physician with myalgia encephalomyelitis/chronic fatigue syndrome. Participants must have access to an electronic, internet connected device to complete the survey. Step 2 – Survey Questionnaire: Participants 18 years of age and older will be asked to complete the survey themselves. Participants younger than 18 years of age or those who do not have the capacity to fill out the survey will have a caregiver complete the survey on their behalf. The survey should take about 30-45 minutes to complete based on piloted data. It is best to be prepared to complete the survey in its entirety in one go, so it is advised that all current supplements and medications be readily available so that brand, type, and dosages can be recorded. However, if this is not possible, you may save your responses and return to the survey within seven days to complete it.

Survey

This survey is intended to examine diet and supplement use in individuals with suspected or diagnosed ME/CFS. Please do not complete the survey if you do not meet this criterion. It is strongly suggested that all supplements, diet products, and medications be readily available when filling out the questionnaire to record type, dose, Natural Product Number (NPN), and Drug Identification Numbers (DIN). As a token of our appreciation, a neck gaiter will be offered to participants. You can request a copy of this consent form for your records and reference.

Please Click One:

○ I am 18 years of age or older and I consent to participate. I meet the criteria and will be filling out this survey for myself. (1)

○ I am 18 years of age or older and I consent to participate. I will be filling out this survey on behalf of a child diagnosed with myalgic encephalomyelitis/chronic fatigue syndrome. (2)

○ I am 18 years of age or older and I will be filling out this survey on behalf of another adult (18 years or older) diagnosed with myalgic encephalomyelitis/chronic fatigue syndrome who is not capable of filling out the survey themselves. (3)

○ No, I do not wish to participate and would like to exit the survey. (4)

1. DEMOGRAPHIC INFORMATION

Q1 If you are completing the questionnaire on behalf of a patient, what is your relationship to them?

○ Parent/Legal Guardian (1)

○ Spouse/Partner (2)

○ Other Family Member (3)

○ Primary Caregiver (e.g., nurse, nanny) (4)

○ Other (Please Specify): (5) ____

○ Not Applicable/I am filling out the survey myself (6)

Q2 Has the participant been diagnosed with myalgic encephalomyelitis/chronic fatigue syndrome by a healthcare provider?

○ Yes (1)

○ No (2)

○ Suspected (Please specify; e.g., history, symptoms, exclusion of other disorders): (3)
____

Q3 Age at diagnosis:

____

Q4 Participant's sex assigned at birth:

○ Male (1)

○ Female (2)

○ Intersex (3)

○ Prefer not to say (4)

Q5 What is the **current** age of the participant?

____

Q6 What is the participant's date of birth? Please write as MONTH, DAY, YEAR (e.g., January 6, 2014).

____

Q7 Participant's current place of residence:

○ Canada (Please specify Province): (1)
____

○ United States of America (Please specify State): (2)
____

◯ Other Country (Please Specify): (3)
____

Q8 Participant's Ethnicity:

◯ Caucasian (1)

◯ Asian/Pacific Islander (2)

◯ Hispanic or Latino (3)

◯ Black or African American (4)

◯ First Nations or Metis or Inuit (5)

◯ Multiracial/Other (Please Specify): (6)
____

◯ Prefer not to say (7)

Q9 If the participant is **under the age of 18 years**, what is the highest level of education **completed by their parent/caregiver?**

◯ High School Diploma (1)

◯ Trade, Technical, Vocational School, or Business/Community College (2)

◯ College Diploma (3)

◯ University Undergraduate Degree (4)

◯ Master's Degree (5)

◯ Professional Degree (e.g., M.D., J.D., D.D.S., Pharm. D.) (6)

- Ph.D. (7)
- Other (Please specify): (8) ____
- Not Applicable/Participant is over the age of 18 (9)

Q10 If the participant is **over the age of 18 years**, what is the highest level of education **completed by the participant?**

- High School Diploma (1)
- Trade, Technical, Vocational School, or Business/Community College (2)
- College Diploma (3)
- University Undergraduate Degree (4)
- Master's Degree (5)
- Professional Degree (e.g., M.D., J.D., D.D.S., Pharm. D.) (6)
- Ph.D. (7)
- Other (Please specify): (8) ____
- Not Applicable/Participant is under the age of 18 (9)

Q11 Please select all the suspected cause(s) or trigger(s) for the participant's ME/CFS diagnosis:

- ☐ Infection (Viral or Bacterial) (Please Specify): (1) ____
- ☐ Immune System Problems (Please Specify): (2) ____

☐ Hormonal Imbalances (Please Specify): (3) ____

☐ Physical and/or Emotional Trauma (4)

☐ Genetic (More common in some families) (7)

☐ Unknown (5)

☐ Other (Please Specify): (6) ____

Q12 What are the participant's top five (5) most bothersome clinical symptoms? **Please select up to 5 symptoms from any category:**

☐ No symptoms (1)

☐ HEART PROBLEMS - Chronic shortness of breath (2)

☐ HEART PROBLEMS - Exercise intolerance (3)

☐ HEART PROBLEMS - Fainting/loss of consciousness (4)

☐ GENERAL SYMPTOMS - Temperature instability (heat/cold intolerance) (5)

☐ GENERAL SYMPTOMS - Physical wasting and malnutrition (Cachexia) (6)

☐ GENERAL SYMPTOMS - Growth delay (7)

☐ GENERAL SYMPTOMS - Difficulty gaining weight (8)

- [ ] GENERAL SYMPTOMS - Persistent sore throat (9)

- [ ] GENERAL SYMPTOMS - Post-exertional malaise (10)

- [ ] GENERAL SYMPTOMS - Enlarged/tender lymph nodes in the neck/armpits (11)

- [ ] GENERAL SYMPTOMS - Decline in social, occupational, educational, and/or personal activities (12)

- [ ] NEUROLOGICAL PROBLEMS - Weakness (13)

- [ ] NEUROLOGICAL PROBLEMS - Developmental delay (14)

- [ ] NEUROLOGICAL PROBLEMS - Developmental regression (15)

- [ ] NEUROLOGICAL PROBLEMS - Mental retardation (16)

- [ ] NEUROLOGICAL PROBLEMS - Difficulty with speech (Dysarthria) (17)

- [ ] NEUROLOGICAL PROBLEMS - Headaches/Migraine (18)

- [ ] NEUROLOGICAL PROBLEMS - Difficulties with memory/concentration/focus (19)

- [ ] NEUROLOGICAL PROBLEMS - Sleep that is not refreshing (20)

- [ ] NEUROLOGICAL PROBLEMS - Dizziness when moving from sitting/lying down to standing/sitting (21)

- [ ] HORMONAL PROBLEMS - Low sex hormones (Hypogonadotropic hypogonadism) (22)

- [ ] HORMONAL PROBLEMS - Hypoparathyroidism (23)
- [ ] HORMONAL PROBLEMS - Hypothyroidism (24)
- [ ] MUSCULOSKELETAL - Muscle cramps (25)
- [ ] MUSCULOSKELETAL - Unexplained muscle and/or joint pain (26)
- [ ] MUSCULOSKELETAL - Muscle tightness (Spasticity) (27)
- [ ] GUT PROBLEMS - Gastrointestinal pseudo-obstruction (28)
- [ ] GUT PROBLEMS - Stomach dysmotility (Gastroparesis) (29)
- [ ] GUT PROBLEMS - Intestinal dysmotility (30)
- [ ] GUT PROBLEMS - Diarrhea/Constipation (31)
- [ ] GUT PROBLEMS - Repeated naesea/vomiting (32)
- [ ] GUT PROBLEMS - Premature satiety (33)
- [ ] GUT PROBLEMS - Irritable Bowel Syndrome (IBS) (34)
- [ ] GUT PROBLEMS - Numerous food intolerances (35)
- [ ] PSYCHIATRIC PROBLEMS - Anxiety (36)

☐ PSYCHIATRIC PROBLEMS - Bipolar Disorder (37)

☐ PSYCHIATRIC PROBLEMS - Depression (38)

☐ Other (Please Specify): (39) ____

Q13 Is the participant currently on any **prescribed or over-the-counter medication(s) for their ME/CFS diagnosis?** If "yes," please list the Drug Identification Number (DIN) for each medication the participant is currently taking. *A DIN is an eight-digit number that Health Canada assigns to each drug. It is located on the label of prescription and over-the-counter medications. If you do not know the DIN(s) or do not live in Canada, please write the drug name(s) and dose(s).*

○ Yes (Please specify DIN(s)): (1) ____

○ No (2)

Q14 Is the participant currently on any **prescribed or over-the-counter medication(s) for other medical conditions?** If "yes," please list the Drug Identification Number (DIN) for each medication the participant is currently taking. *A DIN is an eight-digit number that Health Canada assigns to each drug. It is located on the label of prescription and over-the-counter medications. If you do not know the DIN(s) or do not live in Canada, please write the drug name(s) and dose(s).*

○ Yes (Please specify DIN(s)): (1) ____

○ No (2)

SECTION 2. MOVEMENT PATTERNS

Please note that this section is not suggesting that patients with ME/CFS should be engaging in physical movement. Instead, it is to gain a better understanding and appreciation of the wide spectrum of movement abilities and patterns in people with ME/CFS.

Q15 If physical movement is possible, how many hours does the participant engage in physical movement during a **typical week? If physical activity is not possible, please skip this question or write "not applicable."**

____

Q16 If physical movement is possible, what types of movements does the participant engage in during a **typical week?** (e.g., activities of daily living, gardening, house work, grocery shopping, walking, stretching, yoga, etc.). **If not applicable, please skip this question or write "not applicable."**

____

Q17 If walking is possible, how many steps does the participant walk in a typical day? **If not applicable, please skip this question or write "not applicable."**

____

Q18 If physical movement is possible, does it usually improve or worsen symptoms? Please specify which symptoms are typically improved or worsened. **If not applicable, please skip this question.**

○ Improved (Please specify symptoms): (1) ____

○ Worsened (Please specify symptoms): (2) ____

○ Neutral (No change in symptoms) (3)

Q19 If the participant engages in physical activity, do any of the physical activities **positively impact the participant's symptoms**? If so, please specify the activity/activities and symptoms(s). **If not applicable, please skip this question.**

____

Q20 If the participant engages in physical activity, does the participant track their activity using an activity and/or heart rate monitor? (e.g., Apple Watch, Garmin, etc.) **If not applicable, please skip this question.**

○ Yes (Please Specify): (1) ____

○ No (2)

SECTION 3. DIETARY SUPPLEMENTS

For this survey, we define "dietary supplements" as a product that contains a vitamin, mineral, herb or botanical, amino acid, concentrate, metabolite, or other dietary ingredients intended to add further nutritional value to the diet. Supplements are usually designated in Canada by a Natural Product Number (NPN). They can be found in many forms such as tablets, capsules, softgels, liquids, or powders. Examples include multivitamins, supplementary minerals, protein powders, energy drinks, meal replacements, among many others. Restricted diets such as gluten free, casein free, ketogenic (high fat, low carb), and lactose free will be considered in the next section of this survey.

Q21 Is the participant **currently** taking any dietary supplements?

○ Yes (1)

○ No (2)

Q22 Do you perceive supplement use as being safe?

○ Yes (1)

○ No (2)

○ Other (Please Specify): (3) ____

Q23 If the participant is currently taking dietary supplements, please select all the reasons why they are taking supplements:

▢ Not Applicable/Not currently taking supplements (1)

▢ To increase energy levels or decrease fatigue (2)

- [ ] To increase alertness (3)
- [ ] To limit disease impact (4)
- [ ] To remove toxic metabolites (5)
- [ ] To enhance their diet (6)
- [ ] To improve cognitive ability (7)
- [ ] To promote immune s ystem function (8)
- [ ] To enhance motor skills (9)
- [ ] To increase quality/duration of sleep (10)
- [ ] Recommendation by physician or other health professional (11)
- [ ] Recommendation by close friends/famiy (12)
- [ ] To improve gut health (13)
- [ ] Other (Please specify): (14) ____

Q24 If the participant is not currently taking any dietary supplements, please select all the reasons why they are not taking supplements:

- [ ] Not Applicable/Currently taking supplements (1)

- [ ] May be considered harmful (2)
- [ ] Based on suggestion from close friends/family (3)
- [ ] Read in scholarly article (4)
- [ ] Inadequate knowledge/information (5)
- [ ] Too expensive (6)
- [ ] Never suggested by a physician or other health professional (7)
- [ ] Other (Please specify): (8) ____

Q25 What is your primary source of information regarding dietary supplements?

- ○ Social media (1)
- ○ Online support groups (2)
- ○ Media (e.g., news, magazines) (3)
- ○ Published literature (4)
- ○ Friends/family (5)
- ○ Healthcare Professionals (e.g., physician, nurse) (6)
- ○ ME/CFS Organizations (7)
- ○ Dietitian/Nutritionist (8)

○ Other (Please specify): (9) ____

Q26 Please select all supplements the participant is **CURRENTLY TAKING:**

▢ No supplements (1)

▢ Multivitamin (2)

▢ Vitamin A (3)

▢ Vitamin B1 (4)

▢ Vitamin B1 (Thiamin) (5)

▢ Vitamin B2 (Riboflavin) (6)

▢ Vitamin B3 (Niacin) (7)

▢ Vitamin B6 (Pyridoxine) (8)

▢ Vitamin B9 (Folic acid) (9)

▢ Vitamin B12 (Cobalamin) (10)

▢ Vitamin C (Ascorbic acid) (11)

▢ Vitamin D (12)

- ☐ Vitamin E (Tocopherol) (13)
- ☐ Vitamin K (Phytonadione or Phylloquinone) (14)
- ☐ Vitamin H (Biotin) (15)
- ☐ Alpha-lipoic acid (ALA) (16)
- ☐ Acetyl-L-carnitine (17)
- ☐ Coenzyme Q10 Ubiquinol (18)
- ☐ Coenzyme Q10 Ubiquinone (19)
- ☐ Calcium (20)
- ☐ Iron (21)
- ☐ Creatine (22)
- ☐ Carnitine (23)
- ☐ Arginine (24)
- ☐ Taurine (25)
- ☐ Uridine (26)
- ☐ Other amino acid(s) (27)

- [ ] Selenium (28)
- [ ] Phosphorus (29)
- [ ] Magnesium Oxide (30)
- [ ] Succinate (31)
- [ ] Citrate (32)
- [ ] Protein powder (33)
- [ ] Citrulline (34)
- [ ] Folinic acid (35)
- [ ] N-acetylcysteine (NAC) (36)
- [ ] Omega 3 and/or Omega 6 (37)
- [ ] Vitaminized water (38)
- [ ] Energy drinks (e.g., Redbull) (39)
- [ ] Prebiotic (40)
- [ ] Probiotic (41)

☐ Meal replacement (e.g., Ensure, Boost) (42)

☐ Sports drinks (e.g., Gatorade) (43)

☐ Melatonin (44)

☐ Other (Please specify): (45) ____

Q27 On a scale of 1-5, please rate the perceived degree of change that the dietary supplement(s) have made on the participant's **overall well-being:**

○ 1 - Negatively Impacted (1)

○ 2 - Somewhat Negatively Impacted (2)

○ 3 - Neutral (No change) (3)

○ 4 - Somewhat Positively Impacted (4)

○ 5 - Positively Impacted (5)

○ Not Applicable/Does not take supplements (6)

Q28 Has the participant experienced any improvement of **specific symptoms** since taking dietary supplements?

○ Yes (Please list the symptoms): (1) ____

○ No (2)

○ Not Applicable/Does not take supplements (3)

Q29 Which dietary supplement(s) do you feel have had the most benefit in improving overall wellbeing/symptoms?

____

Q30 Has the participant experienced any side effects from supplement use?

○ Yes (1)

○ No (2)

○ Not Applicable/Does not take supplements (3)

Q31 If yes, please select all the side effect(s) experienced:

▢ Nausea (1)

▢ Vomiting (2)

▢ Upset stomach (3)

▢ Diarrhea (4)

▢ Rash and/or Hives (5)

▢ Headaches (6)

▢ Increased sensitivity to light (7)

▢ Itching (8)

- [ ] Anxiety (9)
- [ ] Bleeding/bruising (10)
- [ ] Kidney problems (11)
- [ ] Liver problems (12)
- [ ] Fatigue (13)
- [ ] Increased body odor (14)
- [ ] Dizziness (15)
- [ ] Confusion (16)
- [ ] Muscle cramping (17)
- [ ] Weight gain (18)
- [ ] Sleep disturbances (19)
- [ ] Loss of appetite (20)
- [ ] Low blood sugar (21)
- [ ] Bad smell (22)
- [ ] Other (Please specify): (23) ____

Q32 Has the participant ever met with a dietitian, nutritionist, naturopath, natural nutrition practitioner, herbalist, or other nutrition professional regarding their diet and supplement use?

○ Yes (Please specify type of health professional): (1)
____

○ No (2)

Q33 Are there any supplements you wish the participant could be taking, but cannot access due to financial constraints and/or insurance coverage?

○ Yes (Please specify the supplement(s)): (1)
____

○ No (2)

Q34 Are there any dietary supplements that the participant has taken but were eventually discontinued?

○ Yes (1)

○ No (2)

Q35 If there have been any dietary supplement(s) that were tried but later discontinued, why was the supplement(s) discontinued? Please select all that apply.

▢ High cost (1)

▢ No benefit (2)

▢ Side effects (3)

▢ Fear of harm or adverse events (4)

- [ ] Bad taste/not pleasant (5)
- [ ] Already taking several supplements and/or medications (6)
- [ ] Forgetfulness (7)
- [ ] Other (Please specify): (8) ____
- [ ] Not Applicable/Does not take supplements (9)

Q36 Is the participant affected by ME/CFS able to take all supplements as recommended?

○ Yes (1)

○ Sometimes (Please specify): (2) ____

○ No (Please specify why not): (3) ____

○ Not Applicable/Does not take supplements (4)

Q37 How are the supplement(s) obtained? Please select all that apply:

- [ ] Compounding pharmacy (1)
- [ ] Regular pharmacy (over the counter) (2)
- [ ] Natural store (3)
- [ ] Via Internet ordering (4)

☐ Other (Please specify): (5) ____

☐ Not Applicable (6)

Q38 The cost of dietary supplements associated with ME/CFS on a monthly basis is:

○ $CAD: (1) ____

○ $USD: (2) ____

○ Other Currency: (3) ____

○ Not Applicable/Does not take supplements (4)

Q39 Please indicate the sources of financial coverage to meet dietary supplement needs (Select all that apply):

☐ No Coverage (1)

☐ Private (e.g., work-based insurance) (2)

☐ Special provincial programs (3)

☐ Special national programs (4)

☐ Unsure (5)

☐ Other (Please specify): (6) ____

☐ Not Applicable/Does not take supplements (7)

Q40 What portion of the cost of the dietary supplements is covered by sources of financial coverage?

○ 0% (No coverage) (1)

○ 1-25% (2)

○ 26-50% (3)

○ 51-75% (4)

○ 76-99% (5)

○ 100% (Full coverage) (6)

○ Not Applicable (7)

Q41 What is the stress (financial, emotional, etc.) imposed by meeting supplement needs due to ME/CFS?

○ Not significant (1)

○ Somewhat significant (2)

○ Significant (3)

○ Very significant (4)

○ Overwhelming (5)

○ Not Applicable/Does not take supplements (6)

SECTION 4. DIETARY RESTRICTIONS

Q42 Which of the following best describes the participant's current diet? Select all that apply:

- ☐ No restrictions (1)
- ☐ Tube fed or parenterally fed (2)
- ☐ Gluten free (3)
- ☐ Casein free (4)
- ☐ Lactose free (5)
- ☐ Ketogenic (i.e., high fat, low carbohydrate) (6)
- ☐ High carbohydrate (7)
- ☐ High protein (8)
- ☐ Vegan (9)
- ☐ Vegetarian (10)
- ☐ Paleo (11)
- ☐ Low FODMAP (12)
- ☐ Mediterranean (13)

☐ Restricted/limited due to sensitivities/intolerances (14)

☐ Other (Please specify): (15) ____

Q43 Are there any foods that the participant avoids completely?

○ Yes (Please specify): (1) ____

○ No (2)

Q44 Are there any special diets that the participant has tried but were eventually discontinued?

○ Yes (Please list the diet(s)): (1) ____

○ No (2)

Q45 If "yes," why was the diet(s) discontinued? Select all that apply:

☐ High cost (1)

☐ Habit and/or convenience (2)

☐ No benefit (3)

☐ Side effects (4)

☐ Fear of harm or adverse events (5)

☐ Bad taste/not pleasant (6)

☐ Switched to a different diet (7)

☐ Other (Please specify): (8) ____

☐ Not Applicable (9)

Q46 Is the participant affected by ME/CFS able to meet all special dietary needs as recommended?

○ Yes (1)

○ Sometimes (Please specify): (2) ____

○ No (Please specify why not): (3) ____

Q47 The cost of special dietary needs associated with ME/CFS on a monthly basis is:

○ $CAD: (1) ____

○ $USD: (2) ____

○ Other Currency: (3) ____

○ Not Applicable/No special dietary needs (4)

Q48 Please indicate the sources of financial coverage to meet dietary needs (check all that apply):

☐ No coverage (1)

☐ Private (e.g., work-based insurance) (2)

- ☐ Special provincial programs (3)
- ☐ Special national programs (4)
- ☐ Unsure (5)
- ☐ Other (Please specify): (6) ____________________
- ☐ Not Applicable (7)

Q49 What is the stress (financial, emotional, etc.) imposed by meeting special dietary needs due to ME/CFS?

- ○ Not significant (1)
- ○ Somewhat significant (2)
- ○ Significant (3)
- ○ Very significant (4)
- ○ Overwhelming (5)
- ○ Not Applicable/No special dietary needs (6)

SECTION 5. HEALTH CARE

Q50 Please describe your comfort level in discussing **nutrition in general** with the participant's primary physician:

- ○ Very comfortable (1)

○ Comfortable (2)

○ Neutral (3)

○ Uncomfortable (4)

○ Very Uncomfortable (5)

○ Not Applicable (6)

Q51 Please describe your comfort level in discussing **dietary supplements** with the participant's primary physician:

○ Very comfortable (1)

○ Comfortable (2)

○ Neutral (3)

○ Uncomfortable (4)

○ Very uncomfortable (5)

○ Not Applicable (6)

Q52 If the participant is currently taking supplements, how many of the dietary supplements have been disclosed to their primary physician?

○ None (0%) (1)

○ Some (1-49%) (2)

○ Half (50%) (3)

○ Most (51-99%) (4)

○ All (100%) (5)

○ Not Applicable/Does not take supplements (6)

Q53 If there are dietary supplements that you have not disclosed to your physician, please select all the reasons as to why they were not disclosed:

▢ Too time consuming (1)

▢ Physician lack of knowledge (2)

▢ No benefit (3)

▢ Scared of judgement (4)

▢ Embarrassed about supplement use (5)

▢ Other (Please specify): (6) ____

▢ Not Applicable (7)

Q54 Apart from nutrition and dietary supplements, what other alternative therapeutic strategies does the participant use to manage their ME/CFS symptoms/diagnosis? Select all that apply:

▢ Napping (Please specify number of naps and length of naps during a typical day): (1) ____

▢ Working with a sleep specialist (2)

☐ Avoiding/limiting social interactions (3)

☐ Avoiding/limiting physical exertion (4)

☐ Avoiding/limiting mental exertion (5)

☐ Avoiding/limiting caffeine during the day (6)

☐ Avoiding/limiting caffeine before bed (7)

☐ Counseling and/or psychotherapy (8)

☐ Following a consistent bedtime routine (9)

☐ Use of sleep apnea machine (10)

☐ Using wearable technology (e.g., smart watch) to monitor heart rate and/or blood pressure (11)

☐ Stretching/yoga (12)

☐ Meditation, deep breathing, and/or other relaxation therapies (Please specify): (13)
____

☐ Massage therapy (14)

☐ Acupuncture (15)

☐ Reiki therapy (16)

☐ Modifying daily activities to reduce exertion (e.g., sitting while showering) (17)

☐ Working with other healthcare specialists (Please specify): (18)
____

☐ Other (Please specify): (19) ____

Q55 Given the limited research surrounding ME/CFS, we are asking that participants provide their consent to be contacted in the future. Consent for each new initiative will be required. Do you provide consent to be contacted by email for future studies?

○ Yes, I consent to be contacted for future studies. My FIRST NAME and EMAIL are: (1)
____

○ No, I do not consent to be contacted for future studies. (2)

References

1. Trudeau MSMS, Madden RFRF, Parnell JAJA, Ben Gibbard W, Shearer J. Dietary and supplement-based complementary and alternative medicine use in pediatric autism spectrum disorder. *Nutrients* (2019) 11: doi: 10.3390/nu11081783

2. Madden RF, Lalonde-Bester S, Parnell JA, Trudeau MS, Martin JM, Khan A, Shearer J. Population-based, cross-sectional assessment of dietary patterns and supplement use in mitochondrial disease. *Clin Nutr ESPEN* (2022) 51:461–469. doi: 10.1016/J.CLNESP.2022.06.016
